# LncRNA NONHSAT030515 promotes the chondrogenic differentiation of human adipose-derived stem cells via regulating the miR-490-5p/BMPR2 axis

**DOI:** 10.1186/s13018-021-02757-z

**Published:** 2021-11-06

**Authors:** Qinqin Yang, Jiajia Guo, Zhijing Ren, Bo Li, Haifeng Huang, Zhen Yang

**Affiliations:** 1 Medical College of Guizhou University, Guiyang, 550025 Guizhou China; 2 Department of Orthopedics, Guizhou Provincial People’s Hospital, Guiyang, 550002 Guizhou China

**Keywords:** lncRNA NONHSAT030515, Human adipose-derived stem cells, Chondrogenic differentiation

## Abstract

**Background:**

Chondrogenic differentiation of human adipose-derived stem cells (hADSCs) is important for cartilage generation and degradation. LncRNAs play an essential role in stem cell differentiation. However, the role and mechanism of lncRNA in hADSCs remain unclear. Our previous study showed that miR-490-5p was downregulated during chondrogenic differentiation of hADSCs. In this study, we investigated the effect and mechanism of lncRNA NONHSAT030515 interacting with miR-490-5p on chondrogenic differentiation of hADSCs.

**Methods:**

Alcian blue staining was used to assess the deposition of chondromatrix proteins following chondrogenic differentiation of human adipose stem cells. Immunohistochemistry was used to evaluate the expression of collagenII. TargetScan, miRTarBase and miRDB database analyses were applied to find the miRNA and target genes of lncRNA NONHSAT030515. A dual luciferase experiment was conducted to identify the direct target of NONHSAT030515. pcDNA3.1- NONHSAT030515 transfection and sh- NONHSAT030515 treatment were conducted to verify the role of lncRNA NONHSAT030515 in chondrogenic differentiation. The levels of Aggrecan, SOX9 and COL2A1 were analyzed by qRT-PCR and Western blot assay.

**Results:**

Alcian blue staining, immunocytochemical, qRT-PCR, and Western blot have determined that lncRNA NONHSAT030515 can promote the chondrogenic differentiation of hADSCs. MiR-490- 5p was the direct target of NONHSAT030515, while BMPR2 was the target gene. This result was confirmed by luciferase reporter assay. Up-regulation of NONHSAT030515 promoted BMPR2 protein expression and promoted chondrogenic differentiation, whereas down-regulation of NONHSAT030515 caused completely opposite results.

**Conclusion:**

LncRNA NONHSAT030515 promotes the chondrogenic differentiation of hADSCs through increasing BMPR2 expression by regulating miR-490- 5p.

## Introduction

Osteoarthritis (OA) is a chronic degenerative disease due to the growth of age, overwork, and other factors caused by articular cartilage damage, also called osteoarthrosis [1]. At present, in order to relieve the pain in the clinic, the main treatment methods include symptomatic treatment, arthroscopic joint cavity irrigation, bone marrow stimulation, and autologous cartilage block transplantation [2]. However, the efficiency of these treatments is not very ideal, so it is urgent to explore more effective treatment methods. In recent years, with the rise of tissue engineering, the use of mesenchymal stem cells to treat osteoarthritis has become an ideal therapeutic approach [2]. Studies on the mechanism of differentiation of mesenchymal stem cells into chondrocyte phenotypes and their regulation may help to develop a new therapeutic strategy OA [22]. Human adipose-derived stem cells (hADSCs) are considered to be a more ideal chondrogenic inducer than bone marrow mesenchymal stem cells (BMSCs) because of their easy access to a large number of cells and strong proliferative ability [3]. Aggrecan, SOX9 and COL2A1 have been reported to be significantly up-regulated after chondrogenic differentiation of human adipose-derived stem cells, and they are also markers of chondrogenic differentiation [9].

LncRNAs are a class of non-protein-coding RNAs more than 200nt in length. It is very abundant, accounting for about 80% of the total non-coding RNA [4]. LncRNA has a complex secondary and tertiary spatial structure and can provide multiple sites to bind to proteins, or have dynamic and specific interactions with DNA and RNA through the principle of complementary base pairing, participating in the regulation of gene expression at the epigenetic, transcriptional and post-transcriptional levels [5]. One of the essential mechanisms that play a role in the post-transcriptional level is the interaction between lncRNA and microRNA (miRNA) to regulate related mRNA activity, namely the ceRNA hypothesis [6].

MiRNAs are small, non-coding single-stranded RNAs specific for tissues or developmental stages and play regulatory roles by binding to specific sequences of target genes post transcription. MiRNAs play an important role in the pathogenesis of a variety of human diseases [23]. Some miRNAs have been shown to be involved in cartilage differentiation and degradation [7, 8]. MiR-499 modulated CUGBP2 and MYBO at the post transcription level in stem cell proliferation and differentiation [23]. MiR-140 can exert major effects on the development of OA in vivo [22]. Our previous study showed that miR-490-5p was down-regulated during chondrogenic differentiation [9]. We predicted the possible target genes of miR-490-5p through bioinformatics analysis.

This study will explore some biological functions and mechanisms of lncRNA NONHSAT030515 in regulating chondrogenic differentiation of human adipose-derived stem cells on the basis of previous studies.

## Materials and methods

### Isolation and culture of hADSCs

Fresh adipose tissue was collected from 35-year-old healthy women provided by Guizhou Provincial People’s Hospital (Guizhou, China). The donor signed informed consent. The adipose tissue was put into the sterile flask with PBS and double antibody, and taken back to the laboratory within 2 h. About 10 g of adipose tissue was brought into the petri dish in the ultra-clean table and cleaned with PBS. Then, the blood vessels and connective tissue were cut off in advance, and the adipose tissue was cut into 1 mm × 1 mm fragments. Then, 0.1% I type collagenase was added to double volume, digestion at 37 °C for 45 min, and centrifuged at 1000r/min for 5 min. The undigested adipose tissue was added with new collagenase for further digestion two times. The obtained cells were resuspended in H-DMEM medium (Thermo, USA), passed 200-mesh cell screen and then seeded into a 10 cm culture dish. The cells were cultured in an incubator at 37 °C with 5% CO_2_. The medium was changed for the first time after 24 h.

### Induce of chondrogenic differentiation of hADSCs by microsphere culture

The fourth generation of human adipose-derived stem cells was selected; 1 × 10^6^ fourth-generation human adipose-derived stem cells were selected into a 15mL centrifuge tube, centrifuged at 1000r/min for 5 min, the supernatant was discarded, and then washed twice with PBS. 3 ml chondrogenic induction medium (H-DMEM, 10 ng/ml TGFβ1, 50 μg/ml VC, and 10^–7^ M dexamethasone, 6.25 μg/ml insulin, 1% double antibody, 1%FBS) was added into the cells for induction for 0, 7, 14 and 21 days. 3–4 days half amount of liquid change.

### Alcian blue staining

After induction of adipose-derived stem cells for 0, 7, 14 and 21 days, the medium was sucked, and 40 g/L paraformaldehyde was added to the cell clumps and fixed for 30 min. Dehydrate with ethanol gradient, embed with paraffin, and prepare slices with a thickness of 5um for use. Blot off the fixed solution and wash with PBS three times. The cell masses were washed with 0.1 N HCl for 5 min. 1% Alcian dye overnight. 0.1 N HCl was washed for 3 times, 5 min each time, and the background was removed. The results were recorded under the microscope.

### Immunocytochemical

After induction of adipose-derived stem cells for 21 days, the medium was sucked, and 40 g/L paraformaldehyde was added to the cell mass for fixation for 12 h. Dehydrate with ethanol gradient, embed with paraffin, and prepare slices with a thickness of 5um for use. Normal goat serum was sealed for 30 min. Rabbit anti-COLII (1:1000) was added and stayed at 4 °C overnight. Rewarm at 37 °C and clean three times with PBS. Subsequently, the secondary antibody was added, incubated at room temperature for 1 h, then washed 3 times with PBS. DAB color development lasted for 5-10 min, and the color development was observed under the microscope.

### Cell transfection

The LncRNA NONHSAT030515 expression was conducted by transfection of pcDNA3.1-NONHSAT030515 and sh-NONHSAT030515. The miR-490-5p expression was conducted by transfection of miR-490-5p mimics. They were designed and synthesized by GenePharma (Shanghai, China). The transfection results were performed using Lipofectamine™ 2000 reagent (Life Technologies Corporation, USA) follow the manufacturer’s protocol.

### Dual-luciferase reporter assay

The dual-luciferase assay was used to confirm whether NONHSAT030515 directly targeted miR-490-5p and whether miR-490-5p directly targeted BMPR2. HADSCs were co-transfected with NONHSAT030515 (BMPR2) wild or mutant plasmids, miR-490-5p control, or miR-490-5p mimics. Luciferase activity was assessed 48 h after transfection using a dual-luciferase reporting system (Promega Company).

### Real-time quantified PCR

First, total RNA was extracted from hADSCs using Trizol reagent (Invitrogen, USA). Then cDNA was synthesized using the PrimeScript RT reagent (Invitrogen, USA). RT-qPCR was performed by SYBR® Premix Ex Taq kit (Invitrogen, USA) using GAPDH as an internal control. The expression miRNA using U6 was used as an internal control. The PCR (20ul) included SYBR Green Premix (10ul), cDNA (2ul), forward primer (10uM; 0.8ul), reverse primer (10uM; 0.8ul) DyeII (0.4ul) and H_2_O (6ul). The cycle was as follows: 95 °C for 30 s, followed by 40 cycles of 95 °C for 5 s, and 60 °C for 30 s, 95 °C for 15 s, 60 °C for 30 s, 90 °C for 15 s. The related primers are shown in Table 1. The outcomes were analyzed by 2^−ΔΔCt^ method.

**Table 1:** Primers used in this study

Primer	Sequence (5′-3′)
miR-490-5p-RT	GTCGTATCCAGTGCGTGTCGTGGAGTCGGCAATTGCACTGGATACGAC ACCCACC
U6-RT	AACGCTTCACGAATTTGCGTG
miR-490-5p-qPCR	Forward: TGCGGCCATGGATCTCCA Reverse: CCAGTGCAGGGTCCGAGGT
U6-qPCR	Forward: GCTCGCTTCGGCAGCACA Reverse: GAGGTATTCGCACCAGAGGA
Aggrecan-qPCR	Forward: AGTCCTCAAGCCTCCTGTA Reverse: CCTCCTCACATACCTCCTG

Primer	Sequence (5′-3′)
SOX9-qPCR	Forward: ACCACCAGAACTCCAGCTCCT Reverse: TCTGCGGGATGGAAGGGA
COL2A1-qPCR	Forward: GCTCCCAGAACATCACCTACC Reverse: TGAACCTGCTATTGCCCTCT
NONHSAT030515-qPCR	Forward: TTGGTGTTGATATGGGTA Reverse: CAAAGTTAGGAGGAAGAA
BMPR2-qPCR	Forward: AAATAGCCTGGCAGTGAG Reverse: ATGTGACAGGTTGCGTTC
GAPDH-qPCR	Forward: GACCTGACCTGCCGTCTA Reverse: AGGAGTGGGTGTCGCTGT

### Western blot analysis

Total protein separated from hADSCs using a protein extraction kit (Bio-Rad, USA), and the concentration of total protein was determined using BCA kit (Tianjin, China). Subsequently, the protein (50 μg) was separated by SDS-PAGE. Then, moved to PVDF (Millipo,USA), the membrane was incubated with blocking buffer at room temperature for 1 h. Then, incubate the first antibody GAPDH (Abcam), Aggrecan (Proteintech), SOX9 (Proteintech), COL2A1 (Proteintech), BMPR2 (Abcam) overnight, followed by secondary antibody at room temperature for 1 h. The GAPDH was used internal control. All western blot experiments were repeated at least three times.

### Statistical analysis

In this experiment, all experimental data were expressed as mean ± standard deviation and were processed by SPSS21.0. An independent sample t-test was used to compare the two groups,and a one-way analysis of variance (ANOVA) was used to compare the groups. P < 0.05 between groups was considered statistically significant. Each group was repeated at least 3 times independently.

## Results

### lncRNA NONHSAT030515 is upregulated during chondrogenic differentiation of hADSCs

**Fig. 1:**
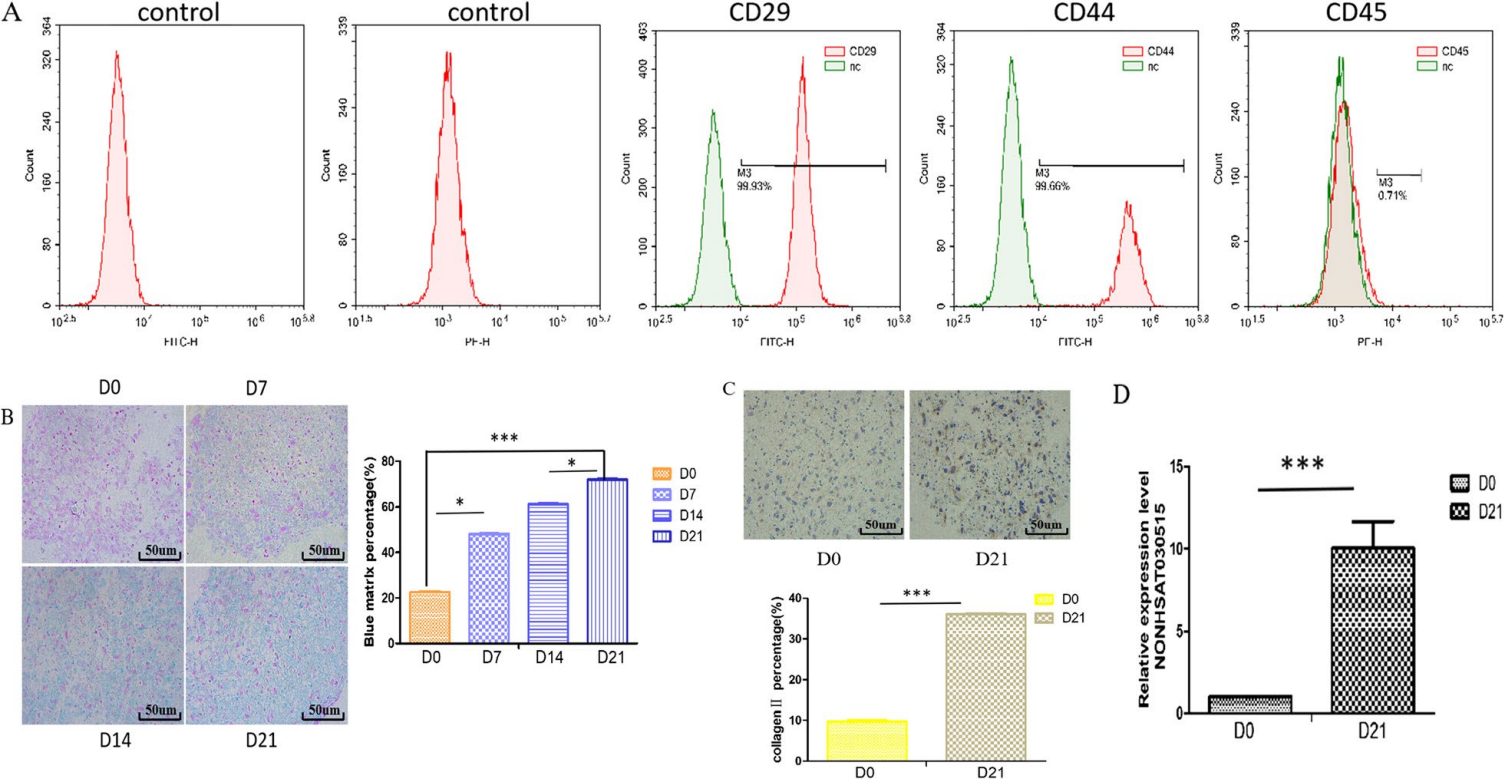
lncRNA NO NHSAT030515 is upregulated during chondrogenic differentiation. **A** Flow cytometric analysis of the surface markers for hADSCs. **B** Alcian blue staining of hADSCs. **C** Immunocytochemical of hADSCs. **D** The expression of NONHSAT030515 was determined by RT-qPCR. ***P < 0.001

To determine the expression level of lncRNA NONHSAT030515 during the chondrogenic differentiation of hADSCs, hADSCs were induced by microsphere culture for chondrogenic differentiation for 0, 7, 14 and 21 days. First, the extracted hADSCs were identified by flow cytometry, and the results showed that CD29 and CD44 were positive, while CD45 was negative (Fig. 1A). Chondrogenic differentiation of hADSCs was assessed by Alcian blue staining, and the deposition of cartilage matrix protein was most obvious after 21 days of induction. Therefore, 21 days of induction was selected as the experimental induction time in subsequent experiments (Fig. 1B). In addition, immunocytochemical results showed an increase in ColII after induction (Fig. 1C). These results indicated that chondrogenic differentiation of hADSCs was successfully induced. Subsequently, the expression of lncRNA NONHSAT030515 after induction was analyzed by RT-qPCR, and the results showed that the expression of lncRNA NONHSAT030515 significantly increased after induction (Fig. 1D).

### lncRNA NONHSAT030515 promoted chondrogenic differentiation of hADSCs

**Fig. 2:**
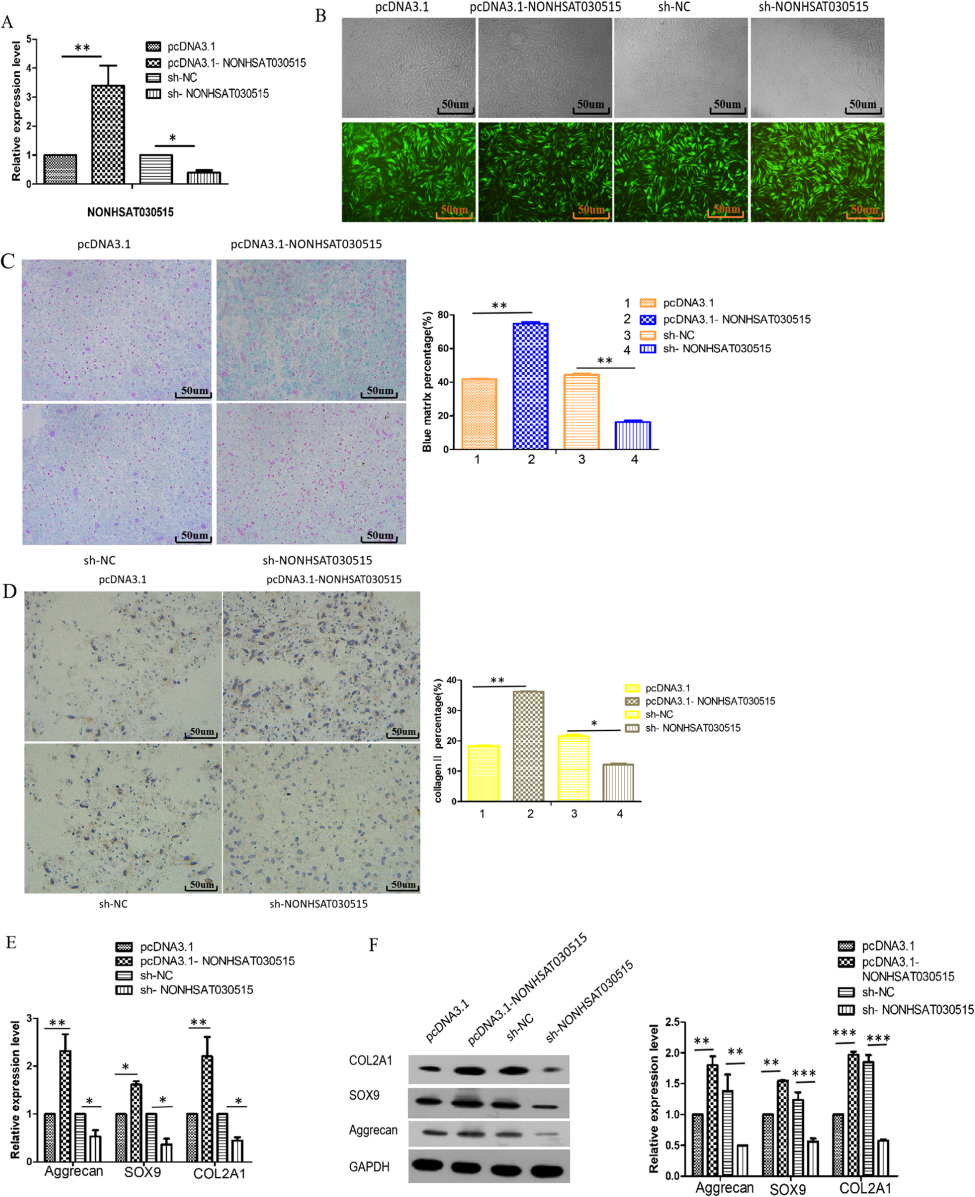
lncRNA NONHSAT030515 promoted chondrogenic differentiation of hADSCs. **A** Expression of lncRNA NONHSAT030515 was determined with RT-qPCR of hADSCs. **B** Infection efficiency was determined by microscopic examination of the GFP-expressing cells. **C** Alcian blue staining of hADSCs. **D** Deposition of collagenII was determined with immunocytochemical of hADSCs. **E** mRNA expression levels of Aggrecan, SOX9 and COL2A1 were detected with RT-qPCR analysis. **F** Protein expression levels of Aggrecan, SOX9 and COL2A1 were detected with western blot analysis. ***P < 0.001, **P < 0.01, *P < 0.05

To investigate the effect of NONHSAT030515 on chondrogenic differentiation in hADSCs, we transfected hADSCs with lentivirus that overexpressed and inhibited NONHSAT030515. RT-qPCR results showed successful transfection (Fig. 2A). GFP-positive cells were observed microscopically, and recombinant the infection efficiency of recombinant lentivirus was detected (Fig. 2B). The GFP-positive cells in each group were about 90%. Alcian blue staining showed increased deposition of cartilage matrix proteins in chondrocytes with pcDNA3.1-NONHSAT030515 (Fig. 2C). Immunohistochemical results showed increased deposition of collagen II in pcDNA3.1 -NONHSAT030515 group (Fig. 2D). In addition, Western Blot and q-PCR experiments showed that the protein expression levels and mRNA expression levels of Aggrecan, SOX9 and COL2A1 were significantly increased in pcDNA3.1-NONHSAT030515 group. It was significantly decreased in sh-NONHSAT030515 group (Fig. 2E, 2F). These data suggested that NONHSAT030515 promoted chondrogenic differentiation of hADSCs.

### miR-490-5p is a direct downstream target of NONHSAT030515

**Fig. 3:**
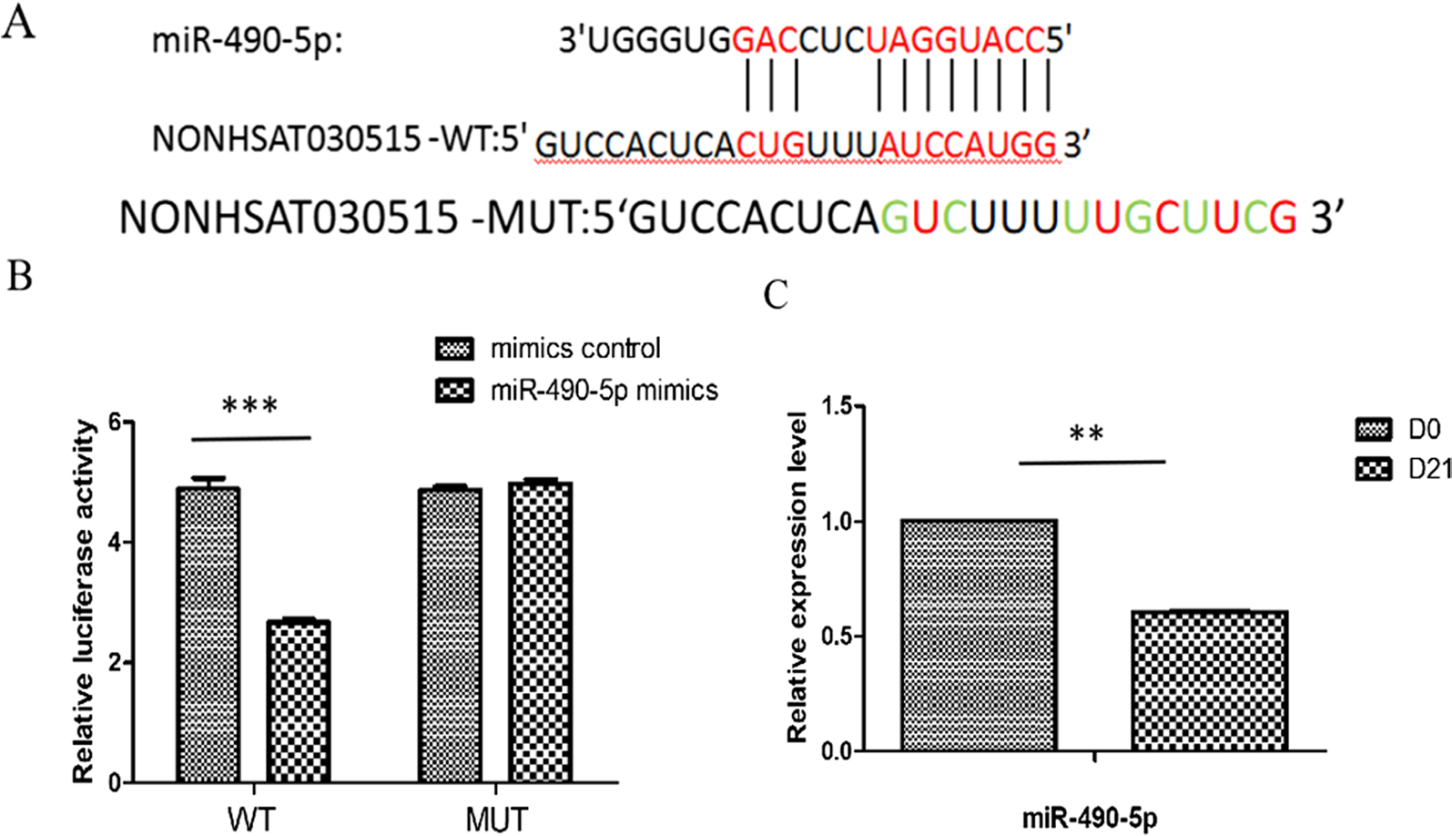
MiR-490-5p is a direct downstream target of NONHSAT030515. **A** The potential binding site of miR-490-5p in the 3'UTR of NONHSAT030515 is shown. **B** hADSCs were transfected with the NONHSAT030515 WT or MUT with either miR-490-5p mimics or miR-490-5p NC, and the luciferase activities were determined using a dual luciferase assay. **C** Expression of miR-490-5p was determined with RT-qPCR of hADSCs. ***P < 0.001, **P < 0.01

Previous studies have shown that lncRNAs play an important role at the post-transcriptional level [6]. In order to investigate the specific mechanism by which NONHSAT030515 promotes chondrogenic differentiation, we used bioinformatics tool (Starbase) to predict potential lncRNA-miRNA binding sites and we found that miR-490-5p targeted binding to NONHSAT030515 (Fig. 3A). We validated the bioinformatics prediction using a dual luciferase assay. The results showed that the luciferase activity of NONHSAT030515-WT was lower than that of the corresponding MUT group, confirming the binding relationship between NONHSAT030515 and miR-490-5p (Fig. 3B). Q-PCR results showed that miR-490-5p was significantly reduced after induction (Fig. 3C). These data suggest that lncRNA NONHSAT030515 directly binds miR-490-5p.

### MiR-490-5p can reverse the effect of lncRNA NONHSAT030515 on chondrogenic differentiation of hADSCs

**Fig. 4:**
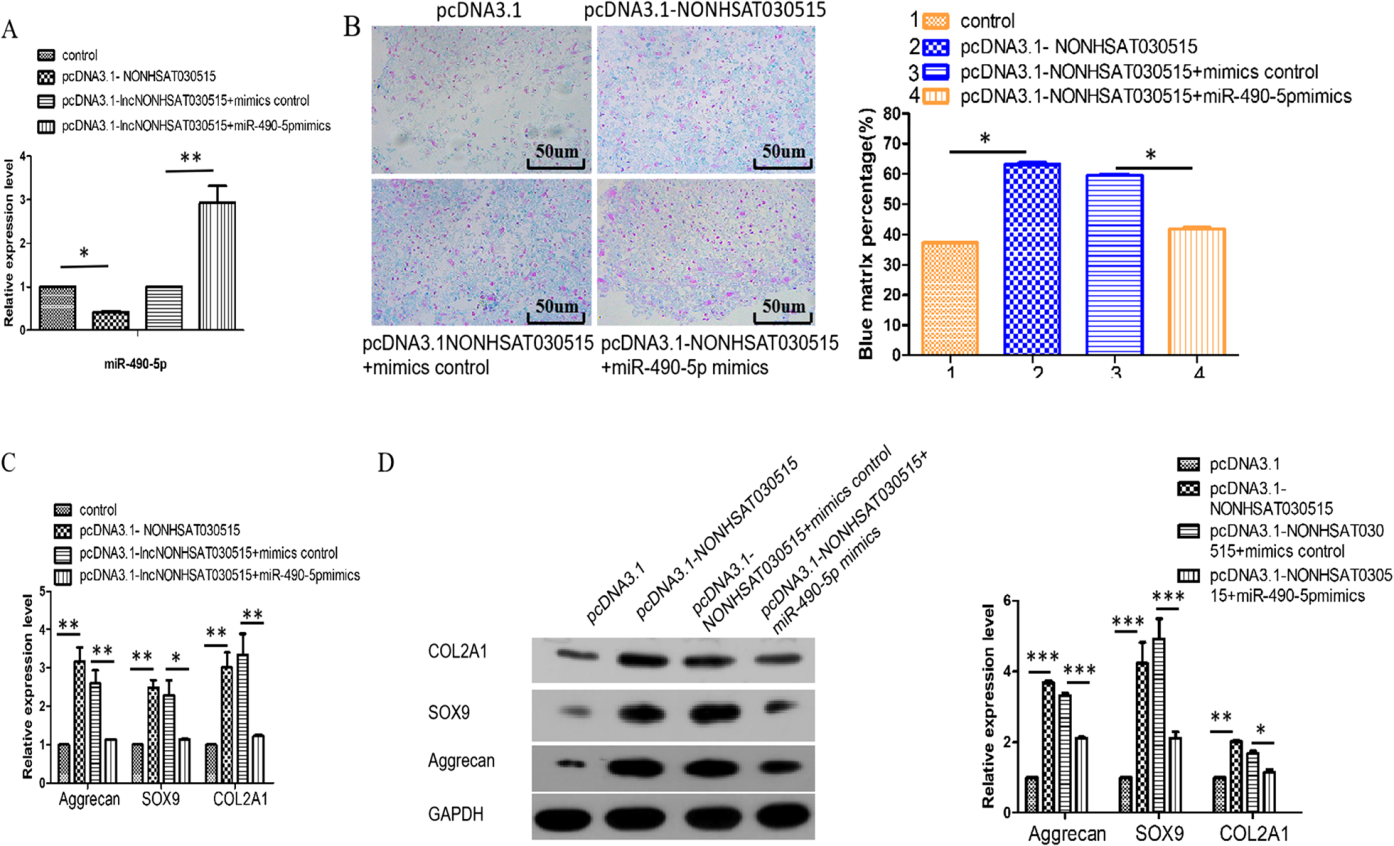
MiR-490-5p can reverse the effect of lncRNA NONHSAT030515 on chondrogenic differentiation of hADSCs. **A** Expression of miR-490-5p was determined with RT-qPCR of hADSCs. **B** Alcian blue staining of hADSCs. **C**, **D** miR-490-5p mimics could reverse the increase in Aggrecan, SOX9 and COL2A1 expression levels caused by NONHSAT030515 overexpression. ***P < 0.001, **P < 0.01, *P < 0.05

In order to confirm the regulatory relationship between lncRNA NONHSAT030515 and miR-490-5p, this was also verified in our study. Compared with pcDNA3.1-NONHSAT030515 + mimics control, the expression of miR-490-5p in pcDNA3.1-NONHSAT030515 + miR-490-5p mimics was significantly upregulated. Compared with pcDNA3.1, the expression of miR-490-5p was significantly downregulated in pcDNA3.1-NONHSAT030515 (Fig. 4A). These results suggested that lncRNA NONHSAT030515 negatively regulated miR-490-5p. In addition, we co-transfected miR-490-5p mimics and pcDNA3.1-NONHSAT030515 in order to investigate whether miR-490-5p inhibited NONHSAT030515 induced chondrogenic differentiation in hADSCs. Alcian blue staining results showed that deposition of cartilage matrix proteins was significantly down-regulated in pcDNA3.1-NONHSAT030515 + miR-490-5p mimics compared to pcDNA3.1-NONHSAT030515 + mimics control (Fig. 4B). What’s more, Western blot and q-PCR experiments showed that the protein expression levels and mRNA expression levels of Aggrecan, SOX9 and COL2A1 were significantly decreased in the pcDNA3.1-NONHSAT030515 + miR-490-5p mimics group. It was significantly increased in pcDNA3.1-NONHSAT030515 + mimics control group (Fig. 4C, 4D). These results suggested that the up-regulation of miR-490-5p could reverse the deposition of cartilage matrix proteins and the expression of Aggrecan, SOX9 and COL2A1 caused by the overexpression of NONHSAT030515.

### BMPR2 is a direct target of miR-490-5p

**Fig. 5:**
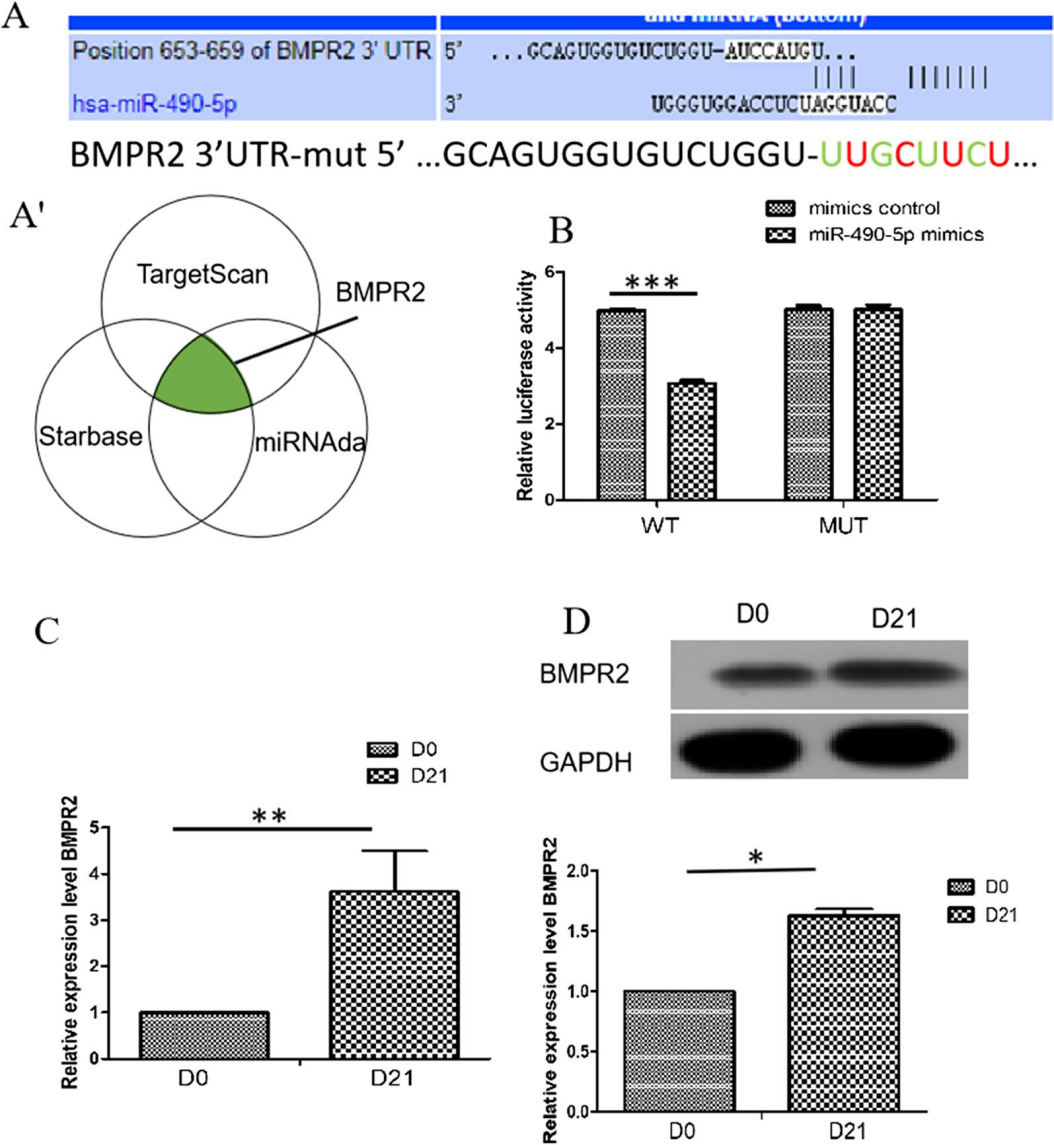
BMPR2 is a direct target of miR-490-5p. **A** The potential binding site of miR-490-5p in the 3'UTR of BMPR2 is shown. **B** hADSCs were transfected with the NONHSAT030515 WT or MUT with either miR-490-5p mimics or miR-490-5p NC, and the luciferase activities were determined using a dual luciferase assay. **C**, **D** Expression of BMPR2 was determined with RT-qPCR and Western blot of hADSCs. **P < 0.01, *P < 0.05

In order to find the potential target genes of miR-490-5p, the prediction was performed by TargetScan, Miranda and Starbase. BMPR2 was the potential target genes of miR-490-5p and the combination sequence is shown in (Fig. 5A). We validated the bioinformatics prediction using a dual luciferase assay. The results showed that the luciferase activity of BMPR2-WT was lower than that of the corresponding MUT group, confirming the binding relationship between BMPR2 and miR-490-5p (Fig. 5B). RT-qPCR and Western blot results showed that BMPR2 was significantly increased after induction (Fig. 5C, 5D). These data suggest that BMPR2 directly binds miR-490-5p.

### BMPR2 can reverse the effect of lncRNA NONHSAT030515 on chondrogenic differentiation of hADSCs

**Fig. 6:**
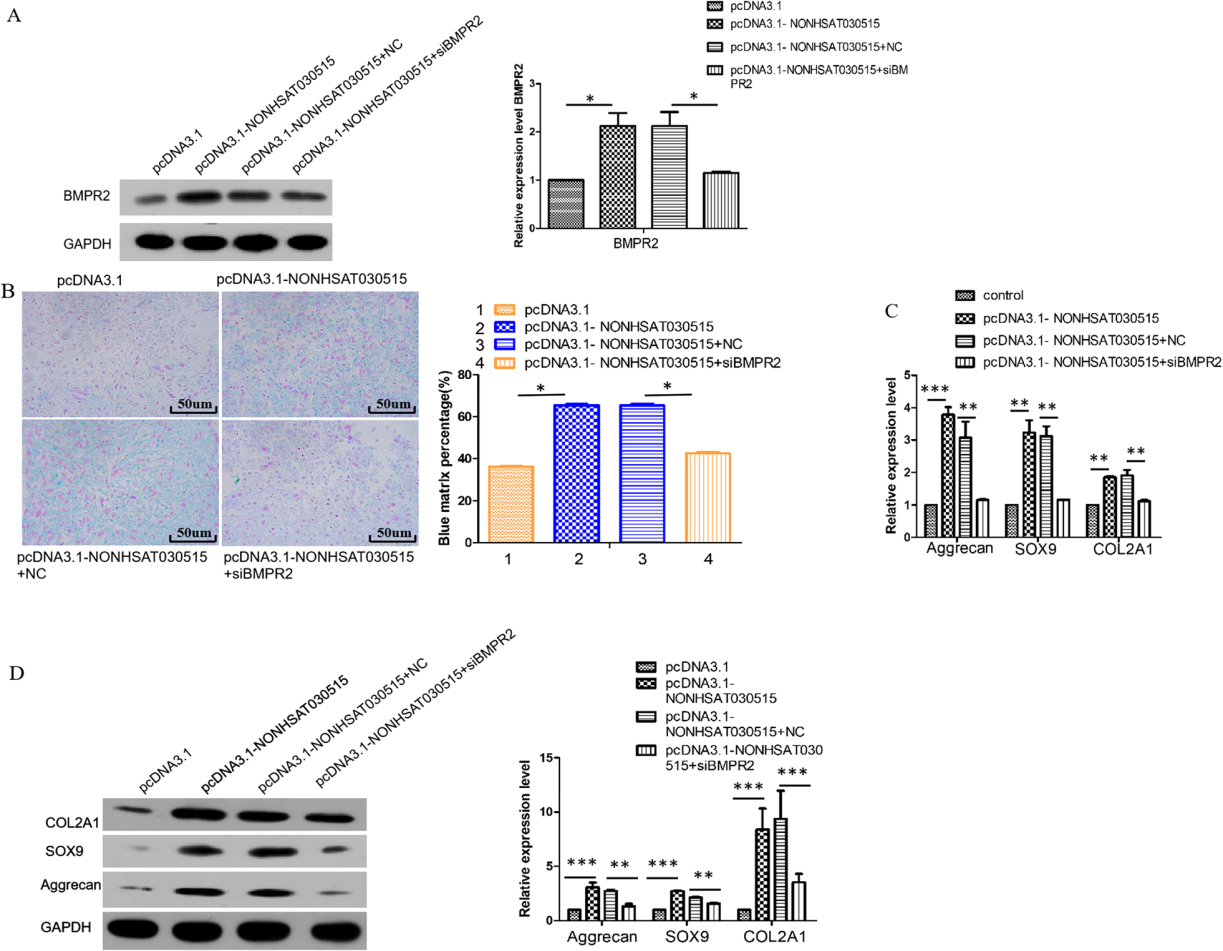
BMPR2 can reverse the effect of lncRNA NONHSAT030515 on chondrogenic differentiation of hADSCs. **A** Expression of BMPR2 was determined with Western Blot of hADSCs. **B** Alcian blue staining of hADSCs. **C**, **D** si-BMPR2 could reverse the increase in Aggrecan, SOX9 and COL2A1 expression levels caused by NONHSAT030515 overexpression. ***P < 0.001, **P < 0.01, *P < 0.05

In order to investigate the effect of NONHSAT030515 on BMPR2, pcDNA3.1-NONHSAT030515 and siBMPR2 were transfected into hADSCs and evaluated by Western Blot. Small interfering RNA plays an important role in exploring the role of genes in mesenchymal stem cells [21].As shown in Fig. 6A, the overexpression of NONHSAT030515 significantly increased the expression level of BMPR2. Alcian blue staining showed increased deposition of cartilage matrix proteins from overexpressed NONHSAT030515 (Fig. 6B). In addition, we also detected the expression of cartilage differentiation markers Aggrecan, SOX9 and COL2A1 by downregulating BMPR2. As shown in Fig. 6C, 6D, siBMPR2 can reduce the mRNA and protein expression levels of Aggrecan, SOX9 and COL2A1 induced by NONHSAT030515. These data suggested that BMPR2 is critical for the function of NONHSAT030515 in chondrogenic differentiation of hADSCs.

## Discussion

**Fig. 7:**
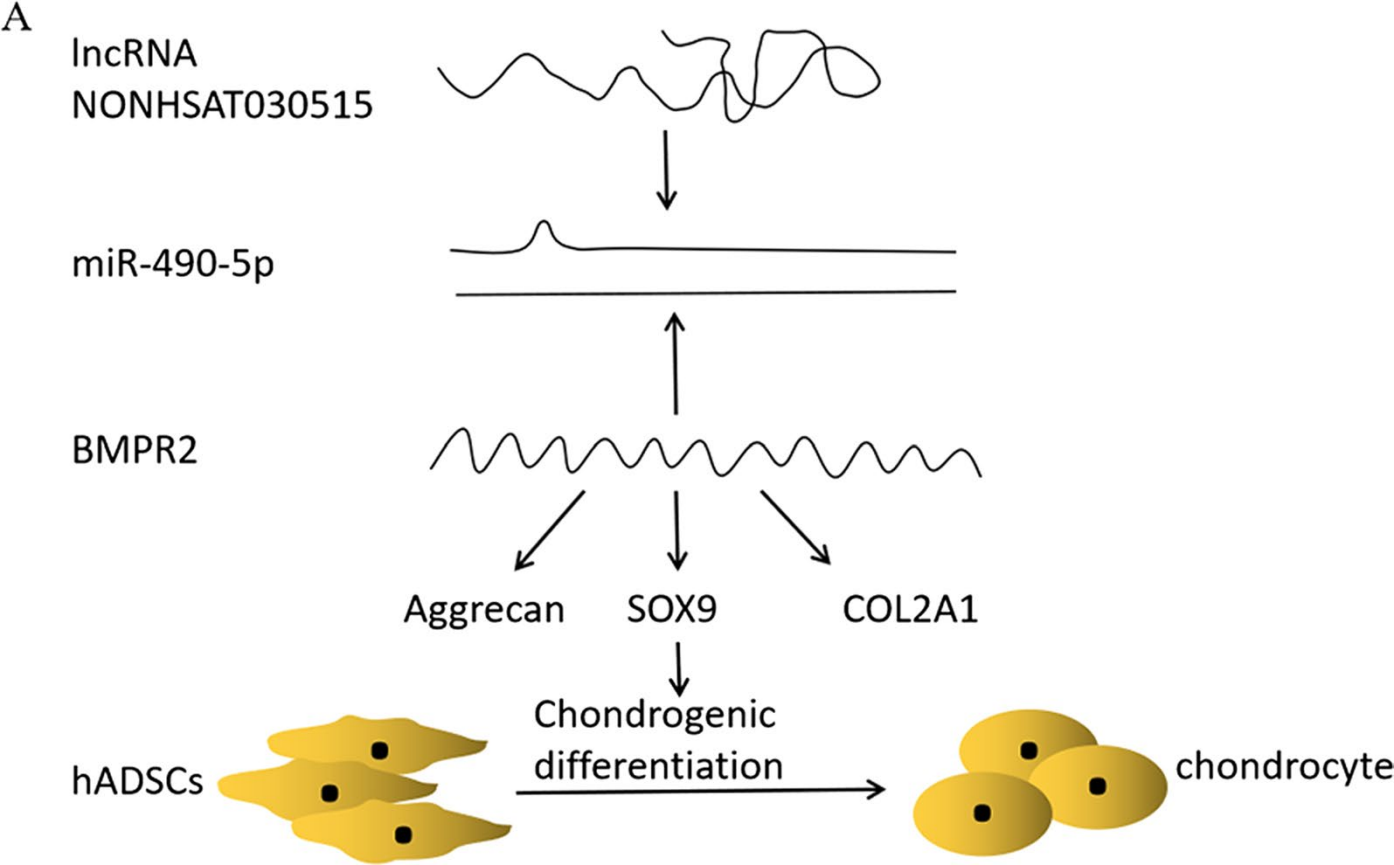
NONHSAT030515 and BMPR2 competitively bind miR-490-5p to regulate downstream Aggrecan, SOX9 and COL2A1, thereby modulating chondrogenic differentiation of hADSCs

In previous studies, we demonstrated that the expression level of miR-490-5p was significantly reduced in chondrogenic differentiation of hADSCs [9], however, the specific mechanism of its interaction with lncRNA is unknown. In this study, we investigated a network axis composed of lncRNA NONHSAT030515, miR-490-5p, and BMPR2, which is involved in regulating chondrogenic differentiation of hADSCs. NONHSAT030515 acts as the miR-490-5p ceRNA to up-regulate the mRNA and protein expression levels of downstream BMPR2. MiR-490-5p inhibited chondrogenic differentiation by decreasing the mRNA expression and protein levels of Aggrecan, SOX9, and COL2A1 and the deposition of collagenII in hADSCs (Fig. 7A).

By pairing with the 3 'UTR base of target mRNA, miRNA inhibits the translation of mRNA or directly degrades mRNA, thus silencing genes at the post-transcriptional level [4, 20]. A growing body of evidence shows that the regulation mechanism of miRNA regulatory genes is realized by regulating the gene expression level during the differentiation of various mesenchymal stem cells.

In recent years, more and more studies have shown that the role of lncRNAs in the form of ceRNAs is involved in a variety of physiological and pathological processes such as cell differentiation, body development and tumor [6]. For example, Wang et al. found that lncRNA MEG3 was up-regulated in BMSCs of mice and patients with postmenopausal osteoporosis, and further studies confirmed that MEG3 inhibited chondrogenic differentiation through direct binding and negative regulation of miR-133a-3p [10]. However, it promotes chondrogenic differentiation of adipose mesenchymal stem cells by binding with miR-140-5p [11]. Recent studies have found that some lncRNAs play important regulatory roles in the chondrogenic differentiation of stem cells from various sources, especially mesenchymal stem cells [12, 13]. For example, a recent study found that lncRNA ROCR promotes chondrogenic differentiation of mesenchymal stem cells by promoting SOX9 expression [14]. Zhang et al. found that lncRNA DANCR promoted chondrogenic differentiation of synovial-derived mesenchymal stem cells by regulating the miR-1305-Smad 4 pathway and the expression of Smad3 and Stat3, and SOX4 [15–17] could activate the expression of DANCR. Carlson et al. reported that lncRNA-HIT could regulate the process of chondrogenic differentiation at the epigenetic level through the recruitment of the p100/CBP complex [18]. However, the regulation of lncRNAs on chondrogenic differentiation of hADSCs has not been reported.

Our study found that NONHSAT030515 promoted chondrogenic differentiation and miR-490-5p reversed the effect of NONHSAT030515 on chondrogenic differentiation of hADSCs. NONHSAT030515 was also confirmed to be the ceRNA of miR-490-5p. In addition, miRNAs are known to regulate gene expression by targeting mRNA 3′UTR [6]. Through bioinformatics analysis, BMPR2 was found to be the target of miR-490-5p. The transcription factor BMPR2 plays a vital role in the chondrogenic differentiation of hADSCs. BMPR2 can reverse the effect of NONHSAT030515 on the chondrogenic differentiation of hADSCs.

This study illustrates the molecular interactions among lncRNA NONHSAT030515, miR-490-5p, and BMPR2 in hADSCs. Therefore, NONHSAT030515 may have potential application value in promoting the chondrogenesis of hADSCs, such as the clinical treatment of osteoarthritis [19]. However, due to the variety of mechanisms through which lncRNA acts and the variety of downstream mRNAs of miR-490-5p, NONHSAT030515 may also cooperate with other molecules to exert its function during chondrogenic differentiation of hADSCs. This requires further research.

## Data Availability

You may access the datasets applied in this study from the corresponding authors upon reasonable request.

## Abbreviations

hADSCs: Human adipose-derived stem cells
miRNAs: MicroRNA
WT: Wild type
MUT: Mutant type
